# Supplementary material for: “Fishing and Hunting”—Selective Immobilization of a Recombinant Phenylalanine Ammonia-Lyase from Fermentation Media
Source: Molecules. 2019 Nov 15;24(22):4146. doi: 10.3390/molecules24224146 (PMC6891789; doi:10.3390/molecules24224146)
Supplement: Supplementary file 1 [file molecules-24-04146-s001.pdf]

molecules

Supplementary Materials

Evelin Sánta-Bell [1], Zsófia Molnár [1,2,3], Andrea Varga [4], Flóra Nagy [1], Gábor Hornyánszky [1,5], Csaba Paizs [4], Diána Balogh-Weiser [1,5,6,*] and László Poppe [1,4,5,*]

[1] Department of Organic Chemistry and Technology, Budapest University of Technology and Economics, 1111 Budapest, Hungary
[2] Fermentia Microbiological Ltd., 1405 Budapest, Hungary
[3] Institute of Enzymology, Research Center for Natural Sciences, Hungarian Academy of Science, 1117 Budapest, Hungary
[4] Biocatalysis and Biotransformation Research Centre, Faculty of Chemistry and Chemical Engineering, Babeş-Bolyai University of Cluj-Napoca, 400028 Cluj-Napoca, Romania
[5] SynBiocat Ltd., 1172 Budapest, Hungary
[6] Department of Physical Chemistry and Materials Science, Budapest University of Technology and Economics, 1111 Budapest, Hungary
* Correspondence: dweiser@mail.bme.hu (D.B.W.); poppe@mail.bme.hu (L.P.)

Table of Contents

1. Equipment 2
2. Analytical methods 2
  2.1. Determination of conversion values by HPLC 2
  2.2. HPLC methods to determine the enantiomeric excess (ee) of D-and L-phenylalanine 2
  2.3. Determination of total protein concentration of the crude lysate and the purified *Pc*PAL 3
  2.4. Amino group quantification on the support 3
3. Experimental methods 3
  3.1. Preparation of ethylenediaminetetraacetic dianhydride 3
  3.2. Immobilization scale up 4
  3.3. Activity measurements during the immobilization, estimateing immobilized *Pc*PAL quantity - biocatalytic activity of *Pc*PAL containing solutions, determined by cinnamate formation 4
  3.4. Reusability tests in ammonia elimination reaction of D,L-phenylalanine 5
  3.5. Reusability tests in ammonia addition reaction onto trans-cinnamic acid 6

1. Equipment

The ^{1}H-NMR spectra were recorded on a Bruker DRX-300 (Billerica, MA, USA) spectrometer operating at 300 MHz, signals are given in ppm on the δ scale. The UV-VIS measurements were carried out in a Genesys 2 type spectrophotometer (Thermo Fisher Scientific Inc., Waltham, MA, USA). The high-performance liquid chromatography (HPLC) analyses were conducted with Agilent (Santa Clara, CA, USA) 1200, 1260, and 1100 systems.

2. Analytical methods

2.1. Determination of conversion values by HPLC

In order to determine the conversions of the immobilized *Pc*PAL-catalyzed ammonia elimination and ammonia addition reactions, samples from biotransformations were injected onto a Gemini NX-C18 column (150 × 4.5 mm; 5 µm) and eluted with a mobile phase: NH_4OH buffer (0.1 M, pH 9.0) / MeOH flow rate of 1.0 mL × min^{-1} at 20 °C using gradient elution (10–39%, 12 min). Conversions were calculated from peak area integrations at 220 nm, with use of response factor 8.81 for the *trans*-cinnamic acid ($t_{r\text{-PHE}}$ = 4.8, $t_{r\text{-}trans\text{-CA}}$ = 9.1).

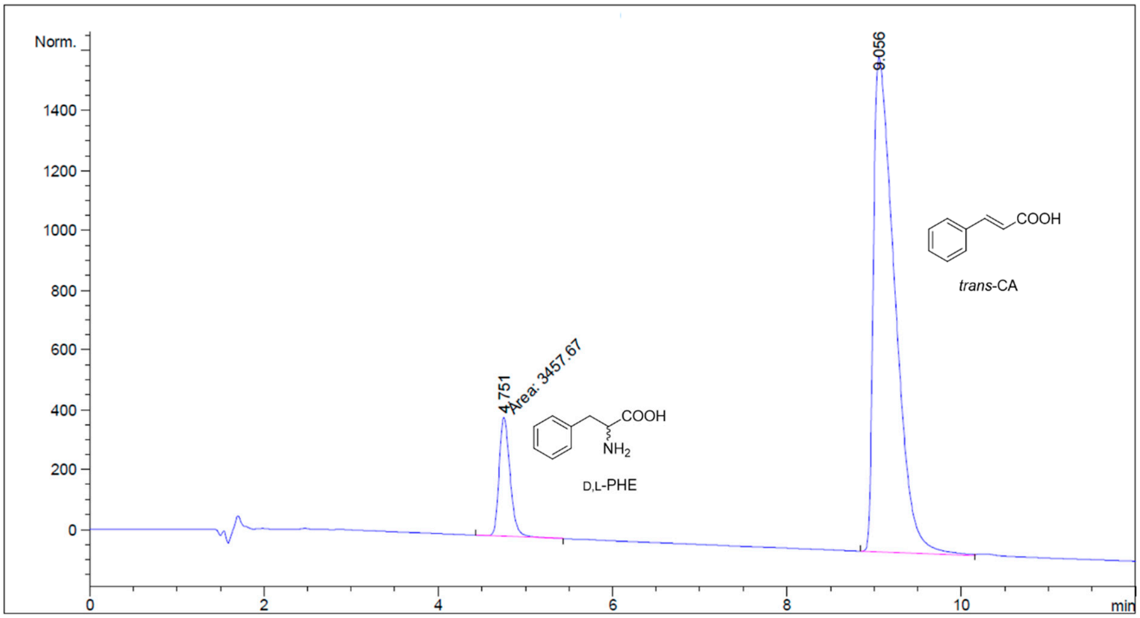

Figure S1. Representative HPLC chromatogram from the separation of D,L-phenylalanine and *trans*-cinnamic acid.

2.2. HPLC methods to determine the enantiomeric excess (ee) of D-and L-phenylalanine

In order to determine the enantiomeric excess, the chiral HPLC separation was performed by using a Crownpak CR-I (+) chiral column (150 × 3 mm; 5 µm) and a mixture of $HClO_4$ solution (pH 1.5) and 20% acetonitrile as eluent, with a flow rate: 0.4 mL·min^{-1} at 15°C ($t_{r\text{-D-PHE}}$ = 3.6, $t_{r\text{-D-PHE}}$ = 6.7).

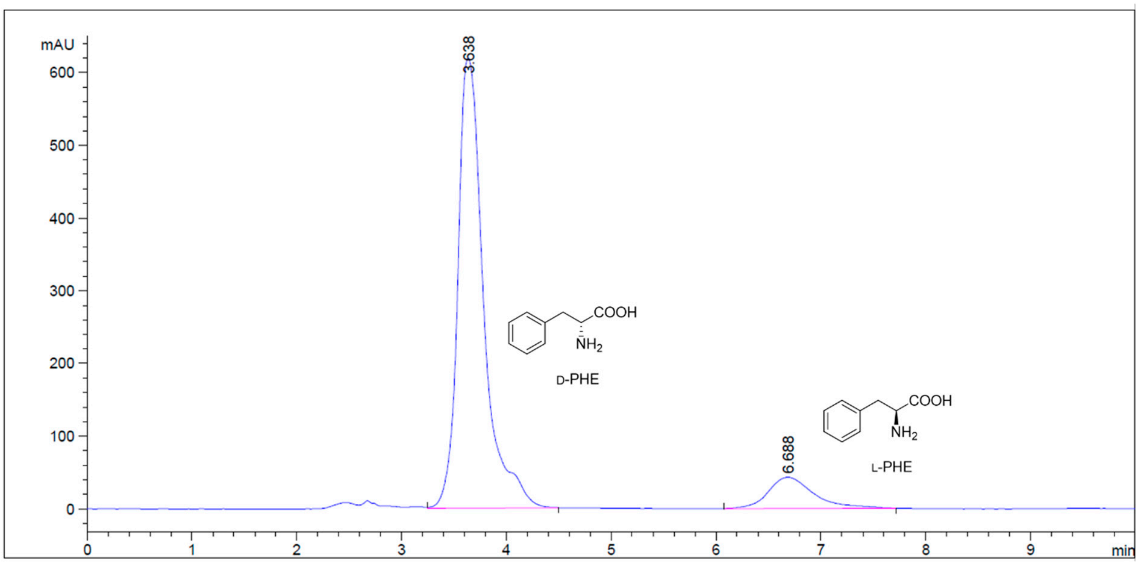

Figure S2. Representative HPLC chromatogram from the separation of D- and L-phenylalanine.

2.3. *Determination of total protein concentration of the crude lysate and the purified PcPAL*

The total protein concentration of the lysate and the concentration of the purified *Pc*PAL were determined with Bradford method. Calibration was performed with bovine serum albumin resulting in a calibration line of $y = 1.22 \times 10^{-4}x - 1.76 \times 10^{-4}$ with $R^2 = 0.986$. In a 1 mL PMMA cuvette, Bradford reagent (900 µL) and tenfold diluted lysate (100 µL) or purified *Pc*PAL (100 µL) were mixed. The UV absorbance was measured at 595 nm after 20 min of reaction time at 30 °C. As an average of three measurements, the whole protein concentration in the cell lysate was 25.6 ± 2.01 g × mL^{-1} containing the target *Pc*PAL in 0.99 ± 0.13 g·× mL^{-1} concentration.

2.4. *Amino group quantification on the support*

Quantification of primary amino functions was carried out by ninhydrin colorimetric assay. The reaction mixture consisted of ninhydrin solution (70 mM in *n*-propanol + 0.2 v/v% acetic acid) and acetate buffer (100 mM, pH 5.5) in 2:1 ratio. For calibration 3-(2-aminoethylamino)-propyldimethoxymethylsilane was used as standard. To the ninhydrin reaction mixture (1.5 mL) silane solution (50 µL, containing the silane in *n*-propanol at 4; 6; 8; 10; 12; 14; 16 or 18 µmol × mL^{-1} concentration) was added and the samples were heated to 95°C for 20 min in 4 mL sealed glass vials. After cooling to room temperature, the absorbance of the solutions was measured at 570 nm, resulting in a calibration line of $y = 4.3715x - 0.4377$ and $R^2 = 0.9899$.

To determine the primary amine content of the magnetic nanoparticles (MNPs) the nanoparticles (4.0–5.0 mg) were added to the reaction mixture (3 mL) and the sample was refluxed for 20 min. After cooling the sample to room temperature, the MNPs were separated with a neodymium magnet and the supernatant was diluted to 5 mL (with *n*-propanol and acetate buffer 2:1 ratio) and the absorbance was measured at 570 nm. As an average of five repeated measurements, the amino group content of MNPs was 360 ± 18 µmol·× g^{-1}.

3. Experimental Methods

3.1. *Preparation of ethylenediaminetetraacetic dianhydride*

Ethylenediaminetetraacetic acid (100 g, 0.34 mol) and pyridine (170 mL) were mixed in a 500 mL four necked flask under argon. The suspension was stirred for 1 h at 65 °C followed by dropwise addition of acetic anhydride (130 mL, 1.34 mol) to the solution. The reaction mixture was kept stirred at 65 °C for 16 h. After cooling to ambient temperature, the resulted solid product was filtered off and

was washed with pyridine (100 mL) and diethyl ether (2 × 100 mL) and dried under vacuum. The white solid product was stored under argon in a refrigerator. Yield: 90 g (97%), ^{1}H-NMR (DMSO-d6): 2.67 (s, 4H, CH2), 3.72 (s, 8H, CH2).

3.2. Immobilization scale up

For the reusability tests the enzyme immobilization was accomplished in larger scale. After metal ion complexation 150–150 mg from the two chosen modified magnetic nanoparticles (MNPs-NPDGE/EDa-10 and MNPs-THPMTGE/EDa-10) was added to the 30–30 ml of crude cell lysate. The immobilization method was the same that described in the article Section 3.4. The immobilized biocatalysts were stored under TRIS buffer (100 mM, pH 8.8) at 4 °C.

In these two case the immobilization selectivity was monitored by the SDS-PAGE (Figure S1). The composition of the covalently attached proteins was estimated with the surface adsorbed proteins after one hour shaking the MNPs samples in the crude protein mixture. After one hour 500 μL samples were taken from the suspensions. The MNPs were separated from the supernatant, washed once time with the lysis buffer and then boiled for 5 min in TRIS buffer in presence of SDS and DTT. Also the supernatant after the immobilization and the washing fractions: low salt (30 mM KCl, 50 mM HEPES, pH 7.5), high salt (300 mM KCl, 50 mM HEPES, pH 7.5), imidazole solution (500 mM in low salt buffer) were examined by SDS-PAGE (Figure S3).

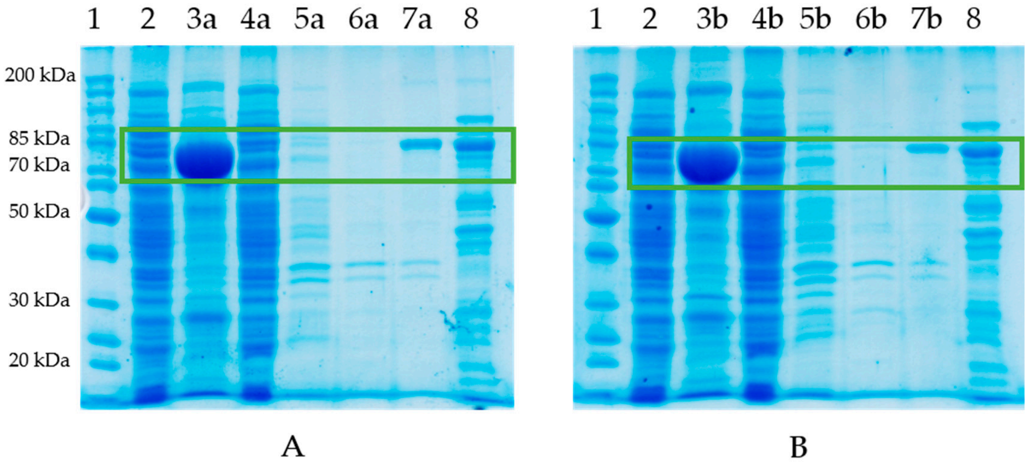

Figure S3. Monitoring the selective immobilization of *Pc*PAL (78 kDa, green framed) onto cobalt contained bifunctional magnetic nanoparticles by SDS-PAGE. a) support: MNPs-NPDGE/EDa-10; b) support: MNPs-THPMTGE/EDa-10. Line 1: Protein marker, line 2: crude cell lysate, line 3: surface adsorbed protein after 1 hour shaking the MNPs in the protein mixture, line 4: supernatant after the 20 h immobilization, line 5: low salt elution fraction, line 6: high salt elution fraction, line 7: 500 mM imidazole elution fraction, line 8: reference *Pc*PAL sample purified by commercially available Speharose-Ni-NTA.

3.3. Activity measurements during the immobilization, estimating immobilized PcPAL quantity - biocatalytic activity of PcPAL containing solutions, determined by cinnamate formation

For calculating the activity yield values the specific enzyme activities were always measured before and after the immobilization as well as the imidazole elution fractions during the washing steps at the end of the immobilization process. The extinction coefficient (ε = 9530 L × mol^{-1} × cm^{-1}) of *trans*-cinnamic acid in TRIS buffer (100 mM, pH 8.8 at 30 °C at λ = 290 nm) was calculated from a calibration line (from 10 points, R^2 = 0.9992). Aliquots (50 μL) of the tested solutions were added to the L-phenylalanine solution (1 mL, 10 mM L-Phe in 100 mM TRIS; pH 8.8) at 30 °C and the time course of *trans*-cinnamic acid formation was recorded at 290 nm for 3 min. Representing these

measurements in case of the two support used in the reusability test the activity curves were shown below (Figure S4).

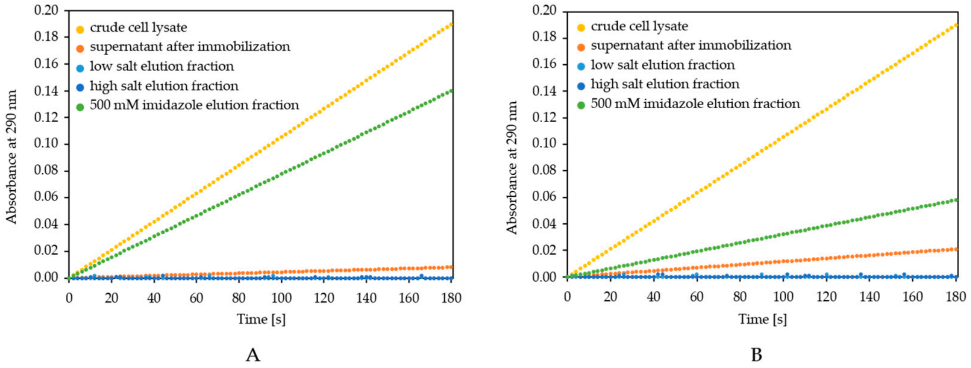

Figure S4. Monitoring the selective immobilization of *Pc*PAL onto cobalt contained bifunctional magnetic nanoparticles (MNPs-NPDGE/EDa-10 (A) and MNPs-THPMTGE/EDa-10 (B)) by activity measurements of the cell lysate before (●) and after the immobilization (●) and the elution fractions: low salt (●), high salt (●) and 500 mM imidazole (●).

The activities in Units were calculated based on the UV-VIS measurements, and the immobilized target protein concentrations were estimated from these data (Table S1).

Table S1. Estimation of immobilized target protein quantity based on the activity values were measured during the immobilization process.

		*Pc*PAL-MNPs-NPDGE/EDa-10	*Pc*PAL-MNPs-THPMTGE/EDa-10
Activity of cell lysate	[U]	0.133	0.133
Activity of supernatant	[U]	0.005	0.015
Activity of imidazole elution fraction	[U]	0.100	0.041
Immobilized activity on 5 mg MNPs	[U]	0.028	0.078
	[%]	21	58
Amount of target protein in 1 ml lysate	[mg]	1.00	1.00
Estimated amount of immobilized target protein on 5 mg MNPs	[mg]	0.21	0.58

The immobilized *Pc*PAL quantity usually were under 10% of the mass of the functionalized MNPs. In some cases, the dried biocatalysts mass was measured back after the test reaction was performed. The deviation from the 5.0 mg (the quantity of MNPs was measured before immobilization) was never greater than the calculated one based on the calculations described above.

3.4. Reusability tests in ammonia elimination reaction of D,L-phenylalanine

In 4 mL screw cap glass vials D,L-phenylalanine solution (2 mL, 10 mM D,L-Phe in 100 mM TRIS, pH 8.8) was added to the *Pc*PAL-MNPs-NPDGE/EDa-10 or the *Pc*PAL-MNPs-THPMTGE/EDa-10 biocatalysts (15 mg wet biocatalysts) and the resulted suspension was shaken at 600 rpm, 30 °C for 5 h. After 5 h samples (50 μL, each) were taken and analyzed by HPLC (for HPLC methods see SI Section 2.1). Between the cycles the samples were washed with TRIS buffer (100 mM, pH 8.8) three times.

3.5. Reusability tests in ammonia addition reaction onto trans-cinnamic acid

In 4 mL screw cap glass vials *trans*-cinnamic acid solution (2 mL, 5 mM *trans*-CA in 6 M ammonia solution adjusted to pH 10.0 with CO_2) was added to the *Pc*PAL-MNPs-NPDGE/EDa-10 or the *Pc*PAL-MNPs-THPMTGE/EDa-10 biocatalysts (30 mg wet biocatalysts) and the resulted suspension was shaken at 600 rpm, 30 °C for 3 h. After 3 h samples (50 μL, each) were taken and analyzed by HPLC (for HPLC methods see SI Section 2.2). Between the cycles the samples were washed with ammonia solution (6 M, adjusted to pH 10.0 with CO_2) three times.
